# Adult-onset leukoencephalopathy with persistent diffusion restriction dot lesions

**DOI:** 10.1007/s10072-023-07212-x

**Published:** 2023-12-08

**Authors:** Di Wu, Jing Zhao, Lan Zheng

**Affiliations:** Department of Neurology, Minhang Hospital, Fudan University, Shanghai, China

## Case details

**Fig. 1:**
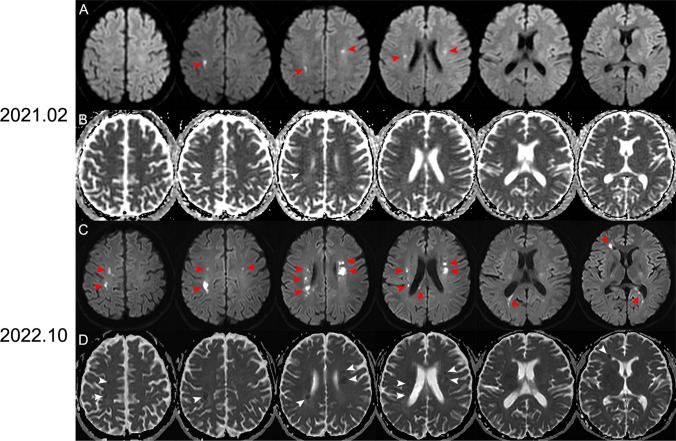
Persistent and deteriorated diffusion restriction dots in deep frontoparietal and periventricular white matter. DWI images (A, C red arrows) showed persistent and deteriorated deep frontoparietal and periventricular white matter diffusion restriction dots, with corresponding reduced ADC (B, D white arrowhead)

A 31-year-old nonconsanguineous man without any obvious clinical manifests exhibited leukoencephalopathy lesions on brain Magnetic Resonance Imaging in a healthy check. No abnormalities were found in the physical examination. Cognitive assessment by MoCA (23/30) revealed mild cognition impairment. Neuroimaging showed persistent and deteriorated symmetric diffusion restriction dots with reduced apparent diffusion coefficient mainly located in frontoparietal and periventricular white matter from February 2021 to October 2022 (Fig. 1). Accessory examination excluded infectious, inflammatory, toxic, neoplastic and acquired demyelinating disorders. Whole exome sequencing confirmed the diagnosis of adult-onset leukoencephalopathy with axonal spheroids and pigmented glia (ALSP), a kind of rare genetic leukoencephalopathy with autosomal dominant inheritance, based on the identification of CSF1R:NM_005211:c.2384 T > C:p.I794T mutation. Persistent diffusion restriction is a characteristic sign raising the possibility of diagnosis of ALSP because it is seldomly existing in other neuroinflammatory or neurodegenerative disorders.[1–3]. Other imaging characteristics of ALSP on MRI are the consistent and confluent symmetric T2 hyperintensities that spare the U-fibers of frontoparietal and periventricular white matter [4].

### Supplementary Information

Below is the link to the electronic supplementary material.

Supplementary file1 (PDF 530 KB)

## Data Availability

The original contributions presented in the study are included in the article/supplementary material, further inquiries can be directed to the corresponding author/s.

## Declarations

The manuscript has not been published elsewhere and is not under consideration by any other journal.

All authors have approved the manuscript.
